# Case Report: Case analysis and literature review of preeclampsia complicated by inevitable abortion progressing to HELLP syndrome with liver rupture and hemorrhage

**DOI:** 10.3389/fmed.2025.1672137

**Published:** 2025-11-05

**Authors:** Guangyang Xing, Yani Lu, Xiaotong Sun

**Affiliations:** ^1^Department of Obstetrics and Gynecology, Gansu Provincial Hospital, Lanzhou, Gansu, China; ^2^The First Clinical Medical College of Gansu University of Chinese Medicine (Gansu Provincial Hospital), Lanzhou, Gansu, China

**Keywords:** HELLP syndrome, diagnosis, treatment, liver rupture, transcatheter arterial embolization, abortion

## Abstract

HELLP syndrome is a severe complication of hypertensive disorders in pregnancy, characterized by hemolysis, elevated liver enzymes, and thrombocytopenia. Severe cases can lead to various complications such as DIC and placental abruption, directly endangering the lives of both the mother and the fetus. Among these, intra-abdominal hemorrhage caused by the rupture of a subcapsular hepatic hematoma is particularly dangerous. Traditionally, the rescue of liver rupture relies on emergency laparotomy. In recent years, the development of interventional radiology has provided a minimally invasive option of transcatheter arterial embolization (TAE) for hemodynamically stable patients. TAE has significant advantages in emergency hemostasis of HELLP-related liver rupture and can reduce maternal and fetal mortality. This case report analyzes a case of preeclampsia complicated with inevitable abortion progressing to HELLP syndrome with liver rupture and hemorrhage. After successful hemostasis with TAE, the patient received comprehensive intensive care including plasma exchange, blood transfusion, and continuous renal replacement therapy. The case emphasizes that early identification, multidisciplinary collaboration, accurate imaging evaluation, TAE for minimally invasive hemostasis, close renal support, and long-term follow-up are the keys to reducing the mortality of HELLP syndrome-related liver rupture and preventing recurrence in future pregnancies.

## 1 Introduction

Hypertensive disorders of pregnancy (HDP) are a leading cause of pregnancy-related morbidity and mortality. They are defined as systolic blood pressure ≥140 mmHg and/or diastolic blood pressure ≥90 mmHg measured on at least two occasions at least 4 h apart. Based on the time of onset and organ involvement, HDP encompasses several diagnoses, including gestational hypertension, preeclampsia, eclampsia, chronic hypertension with superimposed preeclampsia, and chronic hypertension in pregnancy (1). HDP is deficient trophoblast invasion that impairs remodeling of the uterine spiral arteries, precipitating placental ischemia and hypoxia. In response, the ischemic placenta releases anti-angiogenic factors (e.g., soluble fms-like tyrosine kinase-1, sFlt-1) and pro-inflammatory mediators into the maternal circulation, triggering widespread endothelial dysfunction, vasoconstriction, and hypertension. This cascade exposes the mother to acute threats such as eclampsia, HELLP syndrome, placental abruption, multi-organ failure, and death. Simultaneously, reduced placental perfusion forces the fetus into jeopardy—growth restriction, oligohydramnios, preterm delivery, low birth weight, neonatal asphyxia—culminating in stillbirth, neonatal mortality, and long-term neurodevelopmental impairment (2).

Hemolysis, Elevated Liver enzymes, and Low Platelets (HELLP) syndrome is a severe complication of hypertensive disorders in pregnancy, characterized by hemolysis, elevated liver enzymes, and significant platelet decline. This syndrome occurs in approximately 0.2%−0.6% of all pregnancies, but its prevalence rises to 20%−40% among women with severe preeclampsia (3). The maternal mortality rate for HELLP syndrome ranges from 3.5% to 24.2%, while the perinatal mortality rate ranges from 7.7% to 60% (4, 5). Approximately 70 % of HELLP syndrome presents in the late third trimester, whereas 30 % manifests postpartum; the vast majority of postpartum cases emerge within the first 24 h after delivery, with the initial 4 h representing the highest-risk window (6).

Diagnosis of this syndrome relies mainly on hematological examinations. However, due to its symptoms overlapping with other gestational diseases (such as acute fatty liver of pregnancy and preeclampsia), misdiagnosis or missed diagnosis often occurs, delaying the optimal treatment window. In high-risk pregnant patients, early monitoring, standardized treatment, and multidisciplinary collaboration play a significant role in improving maternal and fetal outcomes.

This article reports a successful rescue case by the Department of Obstetrics and Gynecology, Gansu Provincial People's Hospital: a patient with HELLP syndrome and liver rupture hemorrhage after inevitable abortion due to gestational hypertensive disorders. Delivery of the fetus and placenta is not the end-point of hypertensive disorders of pregnancy: *de-novo* postpartum preeclampsia, eclampsia, HELLP syndrome, and subcapsular hematoma can still occur even in women who were normotensive before birth (7). The case highlights the importance of early identification and intervention for critical gestational conditions, reflects the challenges in diagnosing and treating HELLP syndrome and its complications, and further emphasizes the key role of multidisciplinary collaboration and precise therapy in improving patient outcomes. We aim to deepen obstetricians' comprehensive understanding of postpartum HELLP syndrome so that early recognition and standardized management of this obstetric emergency can be markedly improved.

## 2 Case description

### 2.1 Case history

A 32-year-old primiparous woman was admitted to the obstetrics department of our hospital on March 14, 2025, due to “25 weeks and 2 days of amenorrhea and elevated blood pressure detected 1 week ago.” Her last menstrual period was on September 18, 2024, and her expected delivery date was June 25, 2025. Six subsequent routine antenatal visits all showed normal BP. During mid-pregnancy she developed dizziness and mild bilateral lower-limb edema. On 20 February 2025 she was admitted for threatened miscarriage, received tocolytic therapy, and was discharged after improvement. On 12 March 2025 proteinuria 2+ and a maximal BP of 158/110 mmHg were noted at an outside hospital; She self-administered oral labetalol 100 mg every 8 h and measured her blood pressure at home three times daily; recordings ranged from 115–156/80–104 mmHg, prompting her presentation to our obstetric department.

### 2.2 Past medical and family history

She denied a personal history of hypertension, diabetes, coronary heart disease, nephritis, or hepatitis, and no relevant family history of these conditions was reported.

### 2.3 Admission examination

Admission vital signs: blood pressure 140/90 mmHg. Echocardiography revealed left-atrial enlargement with mild mitral regurgitation; routine chemistries, liver and renal panels, and coagulation screen were all essentially normal. Complete blood count: WBC 14.3 × 10^9^/L (90 % neutrophils), RBC 4.05 × 10^12^/L, Hb 120 g/L, platelets 211 × 10^9^/L. Treatment was started with antihypertensives, antispasmodics, dexamethasone for fetal lung maturation, and magnesium sulfate. Subsequent BP recordings ranged 121–146/80–94 mmHg; on hospital day 2 the value was 128/80 mmHg, with a single peak of 146/94 mmHg. Early on hospital day 3 (06:40) the blood pressure was 153/93 mmHg.

### 2.4 Disease progression and treatment

At 04:00 on March 17, 2025, the patient experienced irregular vaginal bleeding, with a blood loss of about 200 ml. Hemostasis and tocolytic treatments were immediately administered. At that time, her vital signs were stable, with a BP of 130/82 mmHg. At 08:30 on 17 March 2025 the patient continued to report irregular lower-abdominal cramps accompanied by vaginal bleeding. Obstetric examination: abdomen distended, mild irregular contractions palpable, small amount of dark-red blood in the vagina. Sterile digital exam: cervical canal almost effaced, internal so admitted two fingers. Because an inevitable abortion was diagnosed at a pre-viable gestation complicated by fetal growth restriction, 200 mg mifepristone was administered for induction of labor after informed consent had been obtained from the patient and her family. After full disclosure and consent, misoprostol induction was started; at 11:30 a 510-g stillborn was delivered ROA with perineal protection. Blood loss was about of 500 mL; uterotonics, antibiotics, and fluids were given. Intra-operative blood pressures were stable at 120–130/70–80 mmHg. After an uneventful return to the ward, her BP remained stable for the rest of the day and overnight, with heart rate ranging 85–93 bpm.

At 08:30 on March 18, the patient reported symptoms of blurred vision, fatigue, and abdominal distension after waking up. Physical examination revealed: BP 146/97 mmHg, ecchymosis at the intravenous puncture site in the upper limb, abdominal distension, tenderness throughout the abdomen, more pronounced in the right upper abdomen, tympanic percussion note, positive shifting dullness, and decreased bowel sounds. Specialist examination showed that the uterus contracted satisfactorily, with bloody lochia, minimal in amount, no foul odor, and no redness or swelling in the perineum. Urgent complete blood count showed hemoglobin of 74.0 g/L and platelet count of 29 × 10^9^/L; Biochemical panel: albumin 21.90 g/L, alanine aminotransferase 93.91 U/L, aspartate aminotransferase 570.81 U/L, gamma-glutamyl transferase 64.36 U/L, lactate dehydrogenase 2,640.76 U/L, serum creatinine 317.37 μmol/L, triglycerides 3.34 mmol/L, high-density cholesterol 0.82 mmol/L; Fibrinogen degradation products 27.15 ug/ml. Autoantibody series and anticardiolipin antibody tests were both negative. The patient has normal blood glucose, normal serum amylase, normal serum lipase, and an ultrasound of the liver and pancreas shows no abnormalities. Acute pancreatitis is currently not considered. An urgent color Doppler abdominal ultrasound was performed, showing: large amount of effusion. Ultrasound showed marked ascites, a structurally intact uterus and no hepatic abnormalities; nevertheless, the abdomen remained distended and the hemoglobin continued to fall. Diagnostic paracentesis with drainage was therefore undertaken to determine whether the fluid was blood or an exudate and to direct further investigation of the etiology. At this time, the patient's general condition was extremely poor, with progressive worsening of fatigue, abdominal distension, and blurred vision, and anuria. Considering the rapid progression of the patient's condition after labor induction, as well as the decreased platelet count, decreased hemoglobin level, and abnormal increases in lactate dehydrogenase and liver enzyme levels, HELLP syndrome was highly suspected, and the patient was transferred to the ICU for further diagnosis and treatment.

**Figure 1:**
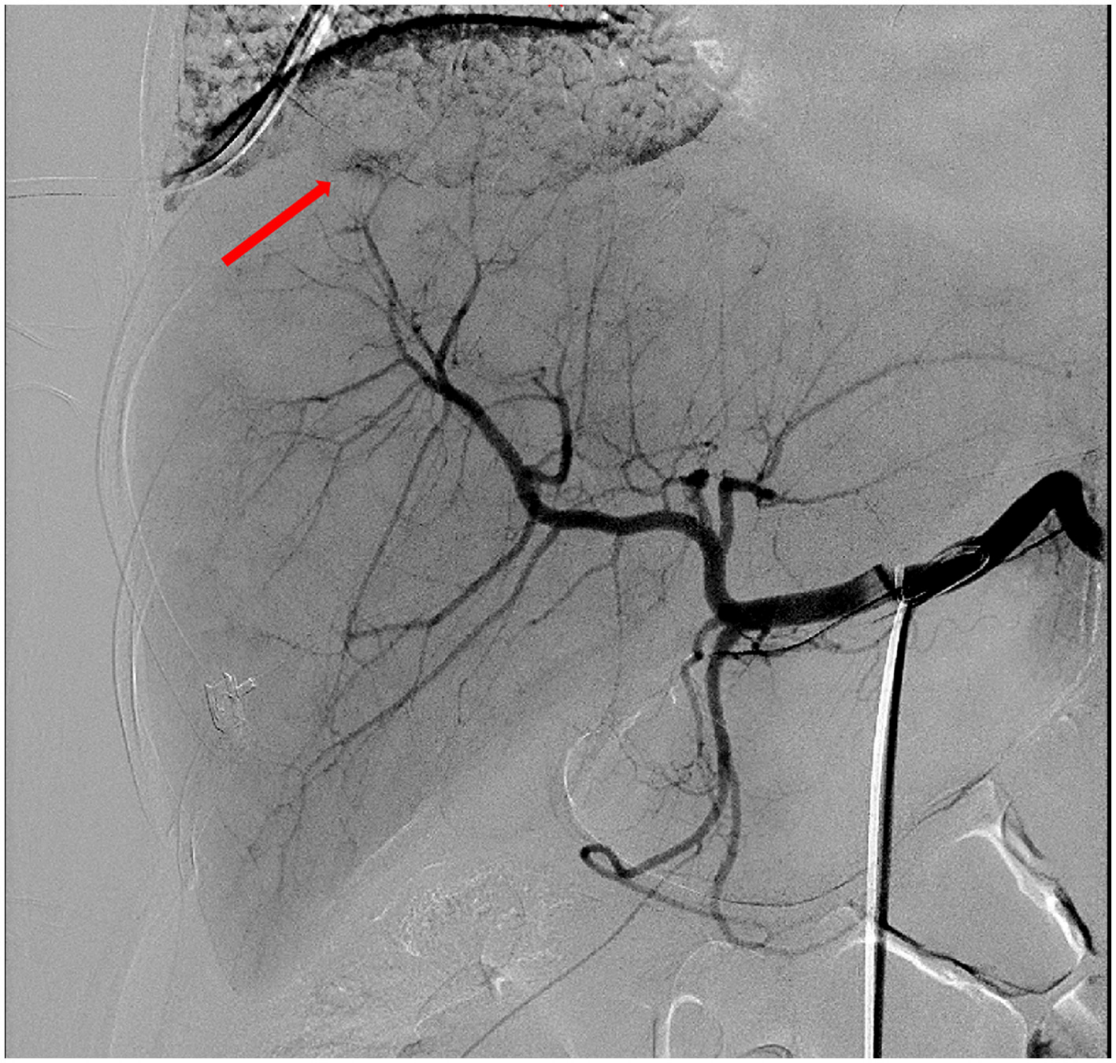
Hepatic artery angiography showed suspected extravasation of contrast agent in the right branch (arrow). Embolization was performed using gelatin sponge injected through a microcatheter, and most of the right hepatic artery was occluded.

On the 1st day after transfer to the ICU, An abdominal puncture and drainage procedure was also performed, draining 1,000 ml of bloody fluid, suggesting the presence of active intra-abdominal bleeding. A repeat complete blood count showed hemoglobin of 40.0 g/L and a platelet count of 30 × 10^9^/L, initially considering the possibility of intra-abdominal bleeding caused by rupture of a subcapsular liver hematoma due to HELLP syndrome. An urgent consultation was requested with general surgery and interventional radiology. Based on the patient's CT findings, it was decided to perform an emergency interventional procedure. During the procedure, hepatic artery angiography revealed suspected bleeding from a right branch vessel. A microcatheter was used to selectively enter the responsible artery, and gelatin sponge was injected through the microcatheter for embolization. A repeat angiography showed good embolization results, with most of the right hepatic artery occluded (Figure 1). Subsequent angiography of the superior mesenteric artery, splenic artery, and uterine arteries revealed no signs of bleeding. The patient successfully underwent transcatheter hepatic artery embolization, with an estimated blood loss of about 1,000 ml during the procedure. Red blood cell suspension was actively transfused to correct anemia. A repeat hemoglobin level rose to 92.0 g/L, indicating significant effectiveness of the blood transfusion and effective control of intra-abdominal bleeding. The subsequent treatment plan included: mechanical ventilation assistance, anti-infection, blood transfusion, nutritional support, albumin supplementation, erythropoiesis and thrombopoiesis promotion, correction of coagulopathy, steroid pulse therapy, continuous renal replacement therapy (CRRT), and five sessions of plasma exchange. After these treatments, the patient's laboratory indicators significantly improved, with liver enzymes returning to normal levels, and her renal function gradually improved, entering the polyuric phase, with multiple repeat renal function tests showing creatinine levels fluctuating around 221 μmol/L. On April 7, 2025, the patient was discharged from the hospital with improved condition. The patient was instructed to continue her oral antihypertensive medication at home and to monitor her blood pressure daily. She was reviewed in the hematology clinic on post-partum day 14: Hb 108 g/L, platelets 227 × 10^9^/L, creatinine 108.44 μmol/L. At the 6-week post-partum visit in the obstetric clinic her blood pressure was normal and complete blood count, liver and renal function had all returned to reference ranges. After 6 months of follow-up she remains well and has resumed full-time work. During a follow-up visit, the patient poignantly shared her mixed emotions: deep gratitude for the care she received, coupled with searing anxiety about embarking on another pregnancy. She reflected that this challenging ordeal had driven home the essential role of meticulous pre-conception planning and vigilant early-pregnancy monitoring.

## 3 Discussion

Hypertensive disorders of pregnancy (HDP) remain one of the leading contributors to maternal and perinatal mortality worldwide, affecting 2–8 % of all pregnancies. Because of their broad impact, high rate of adverse outcomes, and substantial health-care costs, these disorders continue to command global obstetric attention. The 2019 guideline from the American College of Obstetricians and Gynecologists (ACOG) identifies the following major risk factors for gestational hypertension and preeclampsia: prim parity, multiple pregnancy, previous preeclampsia, chronic hypertension, pre-gestational or gestational diabetes, thrombophilia, systemic lupus erythematosus, pre-pregnancy BMI > 30, antiphospholipid-antibody syndrome, advanced maternal age (≥35 years), renal disease, assisted reproductive technology, and obstructive sleep apnea.

### 3.1 Routine identification and clinical manifestations of HELLP syndrome

HELLP syndrome is a rare yet severe pregnancy complication, often regarded as a variant of preeclampsia. Diagnosis typically follows the Tennessee criteria (8): Intravascular hemolysis (laboratory evidence including schistocytes in peripheral blood smear, serum total bilirubin >1.2 mg/dL, hemoglobin decrease, or LDH elevation); Markedly elevated liver enzymes (AST and ALT >2 times the upper limit of normal); Significantly reduced platelets (<100 × 10^9^/L). Fulfillment of all three criteria defines complete HELLP, while thrombocytopenia combined with or indicates partial HELLP (requiring close monitoring). Laboratory features include microangiopathic hemolytic anemia, elevated LDH, and thrombocytopenia, with normal coagulation function (PT/APTT) typically (prolongation suggests DIC). Clinical manifestations mainly include right upper quadrant pain, nausea/vomiting, and fatigue, often accompanied by hypertension/proteinuria, and may secondary to severe complications such as DIC, placental abruption, and renal failure.

The uniqueness of this case lies in the following: the patient only presented with preeclampsia (hypertension, proteinuria) before delivery, without evidence of HELLP; however, after inevitable abortion, she rapidly progressed to typical HELLP manifestations, including fatigue, blurred vision, abrupt platelet drop, hemoglobin decrease, abnormal LDH elevation, liver enzyme abnormalities, and intra-abdominal hemorrhagic ascites. Additionally, the underlying mechanism of HELLP syndrome is associated with placental ischemia/hypoxia releasing inflammatory factors leading to vascular endothelial injury. In this case, threatened abortion and inevitable abortion may have indicated early placental dysfunction, further increasing the patient's risk of progressing to HELLP.

### 3.2 Key points for differential diagnosis

HELLP syndrome requires differentiation from acute fatty liver of pregnancy, autoimmune diseases, thrombotic thrombocytopenic purpura, and hemolytic uremic syndrome, among others.

- **Acute fatty liver of pregnancy**: More common in late pregnancy, liver injury shows obstructive characteristics, with significantly elevated direct bilirubin and blood ammonia, and hypoglycemia (9).
- **Autoimmune diseases**: Exacerbations of systemic lupus erythematosus or antiphospholipid syndrome may also present with hemolysis and thrombocytopenia, but liver enzymes are typically normal or minimally altered, accompanied by clinical features such as lupus erythematosus, arthralgia, and pleurisy. Patients with antiphospholipid syndrome have a history of thrombosis and recurrent preterm birth/miscarriage (especially recurrent miscarriage <10 weeks).
- **Hemolytic-uremic syndrome (HUS)–thrombotic thrombocytopenic purpura (TTP)**: This group of clinical syndromes is characterized by microangiopathic hemolytic anemia and multi-organ damage, presenting with fever, thrombocytopenia, microangiopathic hemolytic anemia, various neurological injuries, and renal damage (10). HELLP syndrome mostly secondary to severe preeclampsia often involves hypertension, with liver as the primary target organ. Clinical manifestations mainly include atypical symptoms such as abdominal pain, nausea, vomiting, and general discomfort; severe hyperbilirubinemia may cause jaundice. Laboratory tests show intravascular hemolysis, elevated liver enzymes, and thrombocytopenia. TTP typically manifests first in the nervous system, and its severity often determines the prognosis (11).

### 3.3 Treatment strategies for liver rupture and hemorrhage

The main maternal complications of HELLP syndrome include subcapsular hepatic hematoma. The pathogenesis may involve HELLP syndrome-preeclampsia stimulating hepatic reticuloendothelial cells, leading to hepatic ischemia, necrosis, and hematoma formation (12). Ultrasonography and CT scans show corresponding findings, with potential liver rupture in severe cases. Routine ultrasound is the initial evaluation method, revealing subcapsular fluid accumulation or hematoma. If feasible, CT/MRI can confirm hepatic lacerations or large hematomas. However, in clinical practice, due to the rapid progression and critical condition of patients, most examinations are performed at the bedside, making accurate diagnosis challenging. Ultrasonography has limitations in diagnosing subcapsular hepatic hematoma rupture: hematomas on the liver surface (e.g., diaphragmatic surface) are easier to detect, but patient mobility restrictions for imaging may hinder diagnosis. In this case, abdominal ultrasound showed no hepatic abnormalities, possibly due to hematoma rupture.

Traditionally, emergency laparotomy has been the rescue measure for liver rupture, involving subcapsular hepatic compression hemostasis, hematoma incision and drainage, or even partial hepatectomy when necessary (13). However, interventional radiology has recently offered new treatment approaches: for patients with relatively stable hemodynamics, TAE can be considered. Reports show that TAE is highly effective in hemostasis and life-saving for HELLP-related liver rupture (14). The patient in this case maintained stable hemoglobin levels without further bleeding after interventional embolization. Arterial embolization may offer swift bleeding control in HELLP-related liver rupture, yet the same endovascular occlusion that stops hemorrhage can convert an already edematous, vasospastic liver into an ischemic target. Potential sequelae—hepatic infarction, infected biloma, sclerosing cholangiopathy, recurrent bleeding, or incomplete embolization—mean that TAE is best regarded as a stabilizing bridge or carefully selected alternative to laparotomy, always contingent on multidisciplinary review and readiness for salvage surgery or transplantation if hepatic ischemia outweighs the gain in hemostasis.

### 3.4 Other comprehensive treatment measures

CRRT has proven to be highly effective in the resuscitation of various diseases, making it an essential supportive measure in the treatment of critically ill patients in the ICU. For patients with severe HELLP syndrome, CRRT treatment should be given in a timely and early manner while providing various symptomatic supportive treatments. This helps to remove various pathogenic factors and metabolic toxins from the patient's blood, correct electrolyte and acid-base balance disorders, and prevent the occurrence of MODS (15). After this patient was weaned off CRRT, there was no significant increase in urine output. It was considered that renal function had not yet recovered and could not meet the body's needs for excreting metabolic waste. After the administration of diuretics intravenously, urine output gradually increased.

Studies have shown that the use of adrenal glucocorticoids can significantly increase the platelet count in patients with HELLP syndrome and reduce LDH and ALT levels (16). Although there is currently insufficient evidence to support the timing, dosage, and duration of adrenal glucocorticoid administration, they should be used early in combination with magnesium sulfate to prevent further deterioration of organ function and promote the recovery of organ function in various systems. Thrombocytopenia is not an absolute contraindication for CRRT. In patients with severe thrombocytopenia who require CRRT, glucocorticoids can be used in combination with plasma and cryoprecipitate transfusions to maintain the patient's coagulation function, allowing CRRT to proceed smoothly and save the patient's life. In the aftermath of the inevitable abortion, the patient developed significant pessimistic emotions. A psychiatric consultation was sought, resulting in a diagnosis of anxiety and depressive state. Management included a combination of pharmacotherapy (anxiolytics and sleep aids) and psychological support, which together stabilized her mood and aided in her psychological convalescence before discharge.

In summary, the patient was successfully treated with a combination of mechanical ventilation, eclampsia management, anti-infection therapy, blood transfusion, nutritional support, albumin supplementation, anemia correction, platelet transfusion, liver enzyme reduction, steroid pulse therapy, CRRT, and five sessions of plasma exchange.

### 3.5 Clinical reflection: early identification, prevention and intervention

Screening and risk assessment:

1. Maintain a high index of suspicion for HELLP syndrome in high-risk pregnancies, particularly in women with a history of early-onset or severe pre-eclampsia, placental dysfunction, or autoimmune diseases.

2. Implement close monitoring of blood pressure, urinalysis, complete blood count, and liver enzymes in these patients, as laboratory abnormalities may precede overt symptoms.

Diagnosis and initial management:

- Hospitalize and initiate close observation for any pregnant patient with suspected HELLP syndrome (e.g., thrombocytopenia, elevated liver enzymes), even in the absence of typical symptoms.
- Extend vigilance to women presenting with “threatened abortion,” as hematological changes may signal an underlying progressive disorder.

Comprehensive and multidisciplinary management:

A proactive, multidisciplinary approach is imperative. For complex cases (e.g., pre-eclampsia with inevitable abortion), collaboration between obstetrics, critical care, hematology, and hepatology is essential to optimize outcomes.

1. Continue close monitoring of clinical and laboratory parameters into the postpartum period to enable timely diagnosis and management of postpartum HELLP syndrome.

## Data Availability

The original contributions presented in the study are included in the article/supplementary material, further inquiries can be directed to the corresponding author.
